# Prospects of Metal‐Free Perovskites for Piezoelectric Applications

**DOI:** 10.1002/advs.202104703

**Published:** 2022-02-24

**Authors:** Han‐Song Wu, Bayu Tri Murti, Jitendra Singh, Po‐Kang Yang, Meng‐Lin Tsai

**Affiliations:** ^1^ Department of Materials Science and Engineering National Taiwan University of Science and Technology Taipei City 10607 Taiwan; ^2^ Graduate Institute of Biomedical Materials and Tissue Engineering Taipei Medical University Taipei City 11031 Taiwan; ^3^ Department of Biomedical Sciences and Engineering National Central University Taoyuan City 32001 Taiwan; ^4^ Graduate Institute of Nanomedicine and Medical Engineering Taipei Medical University Taipei City 11031 Taiwan

**Keywords:** metal‐free perovskites, piezoelectric devices, piezotronics, symmetry breaking

## Abstract

Metal‐halide perovskites have emerged as versatile materials for various electronic and optoelectronic devices such as diodes, solar cells, photodetectors, and sensors due to their interesting properties of high absorption coefficient in the visible regime, tunable bandgap, and high power conversion efficiency. Recently, metal‐free organic perovskites have also emerged as a particular class of perovskites materials for piezoelectric applications. This broadens the chemical variety of perovskite structures with good mechanical adaptability, light‐weight, and low‐cost processability. Despite these achievements, the fundamental understanding of the underlying phenomenon of piezoelectricity in metal‐free perovskites is still lacking. Therefore, this perspective emphasizes the overview of piezoelectric properties of metal‐halide, metal‐free perovskites, and their recent progress which may encourage material designs to enhance their applicability towards practical applications. Finally, challenges and outlooks of piezoelectric metal‐free perovskites are highlighted for their future developments.

## Graphical abstract

In this perspective, the authors summarize the structures and origin of non‐centrosymmetric orientation of metal‐free perovskites. They further discuss the mechanisms including symmetry breaking, external stimulus, and morphotropic phase boundary commonly observed in piezoelectric devices. Finally, they propose possible applications of metal‐free perovskites such as piezoelectric sensors, actuators, nanogenerators, and piezotronic devices.

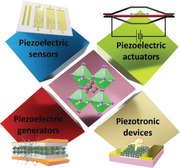

## 1 Introduction

**Figure 1:**
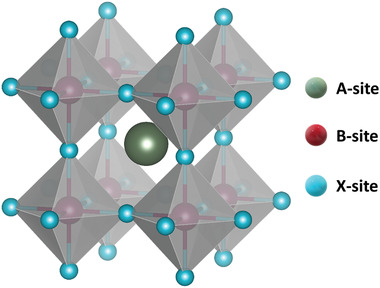
Schematic of the halide perovskite structure.

Since first discovered in 1839, thousands of compounds in the perovskite (ABX_3_, where A and B are cations and X is an anion, respectively) structure shown in **Figure** **1** have been reported.^[^ ^1^ ^]^ Various applications such as capacitors, piezoelectric devices, and ferroelectric devices have been designed by using traditional inorganic perovskite materials, such as barium titanate (BTO, BaTiO_3_) and lead zirconate titanate (PZT, PbZr*_x_* Ti_1−_ *_x_* O_3_).^[^ ^2^, ^3^ ^]^ Recently, metal halide perovskites have attracted wide attention due to their remarkable properties, such as high absorption coefficient, tunable bandgap, high power conversion efficiency, mechanical flexibility, and low‐cost processing.^[^ ^4^ ^]^ These intriguing properties make them potential candidates for various electronic and optoelectronic applications such as diodes, photodiodes, high‐efficiency solar cells, light‐emitting diodes, and sensors.^[^ ^4^, ^5^, ^6^ ^]^ In addition, they have also been studied to exhibit ferroelectric and piezoelectric properties which are comparable to conventional perovskites.^[^ ^3^, ^7^, ^8^, ^9^, ^10^ ^]^ However, the toxicity of the lead component in both conventional PZT and most of metal halide perovskites hinders the development of such materials for practical applications, especially for eco‐friendly electronics. Therefore, the development of lead‐free perovskites has become an urgent matter. Fortunately, a new class of metal‐free halide perovskite materials has been reported to exhibit significant piezoelectricity very recently, making them ideal candidates for future piezoelectric applications.

**Figure 2:**
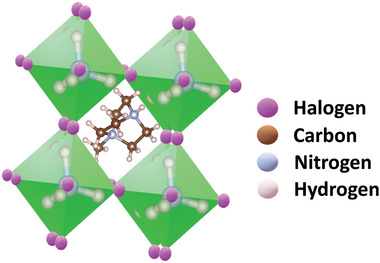
The schematic representation of the metal‐free MDABCO‐NH_4_X_3_ perovskite.

The piezoelectricity is usually observed in materials with non‐centrosymmetric crystal structures which can produce electrical energy upon the external mechanical stimuli or displacement due to the change in the dipole moment within materials. This conversion of energy was first reported in 1880.^[^ ^11^ ^]^ Since then, the remarkable discovery of piezoelectricity has facilitated the fast progress of applications including inkjet printing, injectors, actuators, transducers, sensors, ultrasonic wave detectors, speakers, and nanogenerators.^[^ ^12^, ^13^, ^14^, ^15^ ^]^ Over the past several decades, various piezoelectric materials have also been discovered, such as BTO, PZT, zinc oxide (ZnO), polyvinylidene fluoride (PVDF), peptides, amino acids, and proteins.^[^ ^12^, ^16^, ^17^, ^18^, ^19^, ^20^, ^21^, ^22^ ^]^ Among them, PZT has shown great success in practical applications due to its superior piezoelectric coefficient. However, the lead element within PZT is toxic and can be harmful in the future demands of eco‐friendly and sustainable products. Therefore, lead‐free piezoelectric materials with high piezoelectric coefficient, mechanical softness, and eco‐friendly processing were further investigated.^[^ ^23^, ^24^, ^25^, ^26^, ^27^, ^28^, ^29^, ^30^, ^31^ ^]^ Among them, metal‐free organic halides have gained significant attention due to their promising properties such as low‐temperature processability, biocompatibility, and high piezoelectric coefficient as compared to traditional ceramics.^[^ ^28^, ^29^, ^30^, ^31^ ^]^ The first development of metal‐free piezoelectric perovskite materials can be traced back to 2002, when full organic perovskites, which include C_4_N_2_H_12_·NH_4_Cl_3_·H_2_O with 3D corner‐sharing structure and C_6_N_2_H_14_·NH_4_Cl_3_ with 2D face‐sharing structure have been reported.^[^ ^31^ ^]^ Recently, quasi‐spherical and momentum matching theories have been applied to precisely design a series of metal‐free perovskites with polar structures.^[^ ^32^ ^]^ In these studies, materials including MDABCO‐NH_4_I_3_ (MDABCO = N‐methyl‐N′‐diazabicyclo[2.2.2]octonium) and hmtaH_2_‐NH_4_Br_3_ (hmta = hexamethylenetetramine) have been reported to exhibit non‐centrosymmetric structures that can induce high ferroelectric and piezoelectric constants.^[^ ^33^, ^34^, ^35^ ^]^ The schematic representation of these organic perovskites is shown in **Figure** **2**. Thus, these works show a wide diversity of material combinations in metal‐free perovskites that can be designed to make a polar structure, enabling the control of ferroelectric, piezoelectric, and optical properties for various applications.

To explore the above‐mentioned promising properties of metal‐free perovskites, many strategies have been developed for achieving functional electronic, optoelectronic, and ferroelectric devices. However, the fundamental understanding of piezoelectricity and their piezoelectric device applications is still scarce and challenging. Therefore, in this perspective, we explore the molecular design by understanding the symmetry breaking of metal‐free perovskites. Later, piezoelectric devices and applications due to the control of structure and composition will be discussed. Finally, the potential and challenges of metal‐free perovskites will be highlighted since these novel designs can provide new possibilities to further extend the applications of metal‐free perovskites.

## 2 Design Theories

### 2.1 Symmetry Breaking

**Figure 3:**
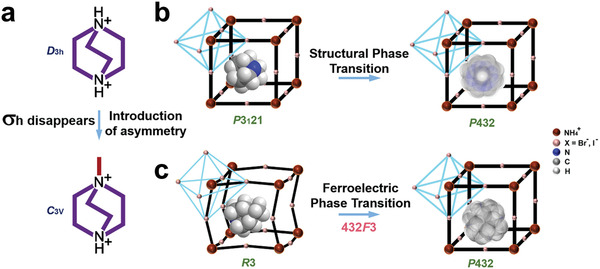
a) Modification of molecular symmetry. b) Initial structural phase transition. c) Modified phase transition. Reproduced with permission.^[^ ^32^ ^]^ Copyright 2019, American Chemical Society.

It has been investigated that the origins of both ferroelectricity and piezoelectricity arise from non‐centrosymmetric crystal structures. A slight displacement of cations can effectively induce structure distortion.^[^ ^36^, ^37^ ^]^ This kind of phenomenon can be observed in the family of oxide perovskites having titanium as the B‐site cation, such as CaTiO_3_, SrTiO_3_, BaTiO_3_, and PbTiO_3_.^[^ ^38^ ^]^ For organic–inorganic hybrid perovskites, it was not until recently that the effect of organic cation has been reported to play a role in the symmetry‐breaking mechanism.^[^ ^39^ ^]^ As shown in **Figure** **3**, the quasi‐spherical theory is a phenomenological theory based on the Curie's symmetry principle.^[^ ^32^, ^35^, ^40^ ^]^ By modifying the spherical cation to a low‐symmetric quasi‐spherical geometry and combining it with the momentum matching of cation and anion, a molecular rotation can be formed at a suitable rate (Figure 3a–c).^[^ ^32^, ^35^ ^]^ In addition, the self‐polarization of the material is dominated by the number of polarization directions.^[^ ^41^ ^]^ Therefore, the symmetry change induced by the quasi‐spherical modification results in the production of multi‐axial materials. The highly ordered dipole directions and dipole switching behaviors in the polycrystalline state become ideal properties for ferroelectric/piezoelectric applications.

### 2.2 External Stimulus

It is very crucial to induce the phase suitable for piezoelectric applications.^[^ ^42^ ^]^ Piezoelectric materials, such as PVDF exhibiting piezoelectricity in *β*‐phase.^[^ ^43^ ^]^ It was reported that electric field or heat can be used to facilitate the formation of *β*‐phase in PVDF‐based piezoelectric generators.^[^ ^44^ ^]^ In addition, by adopting electric field‐induced phase transition or structure distortion, inorganic metal‐halide perovskites such as CsPbBr_3_ can show higher piezoelectric constants. The piezoelectric coefficient (d_33_) increases from 7.7 to 40.3 pm V^−1^.^[^ ^45^ ^]^ In addition, the PbBr_6_ octahedra in the orthorhombic lead bromide can be well‐aligned by applying an electric field.^[^ ^46^ ^]^ Similar strategy can also be applied to 3D metal‐free perovskites to improve piezoelectricity. As for the heat‐induced piezoelectricity, a new type of metal‐free perovskite H_2_DABCO(NH_4_)(BF_4_)_3_ has been synthesized serving as a temperature‐stimulus responsive material (TSRM) due to a low phase transition temperature at ≈332–333 K.^[^ ^47^ ^]^ This type of TSRM can be adopted in specific devices that switches piezoelectric response into different temperatures.

### 2.3 Morphotropic Phase Boundary

To design materials with high piezoelectric response, a morphotropic phase boundary (MPB) is a versatile feature to enhance electromechanical properties of lead‐based and organic lead‐free perovskites.^[^ ^48^ ^]^ MPB materials display transition regions in composition phase diagrams where materials change their lattice structure abruptly with a lower energy barrier of polarization rotation, indicating a high coupling of strain and polarization.^[^ ^49^, ^50^ ^]^ Indeed, MPB is a phase coexistence area in the phase diagram correlated to the competition of the two phases with different orientations of polarization.^[^ ^51^ ^]^ It has been discovered that higher electro‐mechanical coupling efficiency occurs within the MPB to exhibit a large piezoelectric constant (d_33_) due to polarization rotation between two competition phases in perovskites.^[^ ^50^ ^]^ Meanwhile, it should also be noted that some critical factors are required to form a solid solution MPB. The requirements are shown as below:

1. Goldschmidt tolerance factor: a stable perovskite structure should obey the tolerance factor between the range of 0.825–1.059.^[^ ^52^ ^]^ When it comes to determining a composition of solid solution, the tolerance factor can be a tool to evaluate whether the structure will be stable.
2. Solubility: the solubility acts as a key role in forming a solid solution MPB since low solubility will cause phase separation and limit the final composition.^[^ ^53^, ^54^ ^]^
3. Slope of the phase boundary in the phase diagram: the slope of the phase boundary is critical to the phase stability under transition.^[^ ^55^ ^]^ By adjusting the composition, the MPB phase can be stable in a wider temperature range.^[^ ^55^, ^56^ ^]^

Previously, many researchers have focused on tuning the MPB of PZT in which the largest piezoelectric constant can be obtained with a Zr/Ti ratio of 52:48.^[^ ^25^ ^]^ Recently, a halide perovskite molecular solid solution (TMFM)*_x_* (TMCM)_1−_ *_x_* CdCl_3_ has been demonstrated to show a piezoelectricity stronger than PZT.^[^ ^32^ ^]^ However, this type of solid solution faces toxicity issues due to the presence of cadmium ions. Therefore, non‐toxic metal‐free perovskites such as MDABCO‐NH_4_Br, exhibiting a high d_15_ piezoelectric response (d_15_ = 248 pm V^−1^) and A‐site cation variability, have become ideal candidates for developing a non‐toxic halide perovskite molecular solid solution MPB.^[^ ^36^, ^57^ ^]^

### 2.4 Design Route

In general, the toxicity and stability of the perovskite's devices are the two foremost issues to be considered prior to designing eco‐friendly and highly efficient metal‐free perovskites.^[^ ^58^, ^59^, ^60^ ^]^ The fundamental chemical design offers an efficient route by considering the ionic radius and formal charge neutrality, and replacing toxic elements with low/non‐toxic elements.^[^ ^60^, ^61^, ^62^, ^63^, ^64^ ^]^ For instance, Pb^2+^ in lead‐halide perovskite was first replaced by divalent Sn^2+^ and Ge^2+^ cations due to their adequate charge balance and coordination.^[^ ^65^, ^66^ ^]^ Interestingly, first‐principle studies and machine‐learning methods (ML) have been recently employed to predict and provide the accurate prerequisite and rational design of the materials for structurally stable and low‐toxic metal‐free perovskites.^[^ ^60^, ^67^, ^68^ ^]^ The systematic high‐throughput simulation with first‐principle studies (or density‐functional theory [DFT] calculations) offers a plausible tool to design and explore the material's structure and electronic properties particularly to screen the most probable candidates from a large material database.^[^ ^67^, ^69^ ^]^ ML in conjunction with DFT calculations have been employed to efficiently discover inorganic double perovskites structures as well as hybrid (organic–inorganic) halide perovskites (e.g., C_2_H_5_OInBr_3_, C_2_H_5_OSnBr_3_, and C_2_H_6_NSnBr_3_) for stable, non‐toxic, and highly efficient solar cells development.^[^ ^70^, ^71^, ^72^, ^73^, ^74^ ^]^ ML technology has also been applied to design Pb‐free BTO‐based piezoelectrics with large electrostrains.^[^ ^75^ ^]^ ML and DFT could also be useful to evaluate the structure–property relationship of the material candidates such as to map the bandgaps, band structures, and density‐of‐states of the novel materials for highly stable and environmentally friendly metal‐free perovskites.^[^ ^67^, ^76^, ^77^ ^]^ In piezoelectric‐induced metal‐free perovskites, the use of DFT and ML in combination with experimental observation may accelerate the discovery of highly efficient, stable, and low/non‐toxic perovskites materials.

## 3 Properties and Applications

Inorganic materials exhibit piezoelectricity from the displacement of ions inside crystals. As the inorganic piezoelectric materials undergo an external electric field, the atomic structure of the crystal changes in such a way that the balance of ions shifts and a dipole moment is produced. To develop net polarization in the crystal, the generated dipole should not be canceled out with other dipole moments present in the unit cell of the crystal. Therefore, the piezoelectric atomic structure should be non‐centrosymmetric. On the other hand, in the case of organic materials, the piezoelectricity arises due to the reorientation of molecular dipoles within the materials and can be obtained under the application of high electric field and stretching. Recently, various organic materials such as PVDF, glycine, collagen, and silk have been widely investigated. Unlike inorganic piezoelectric materials, PVDF exhibits a negative piezoelectric constant (d_33_) attributed to the self‐consistent quantum redistribution of electron molecular orbitals, the shifting of charged atomic nuclei, and the dipole reorientation upon the application of electric field. Most recently, metal‐free perovskites including C_4_N_2_H_12_‐NH_4_Cl_3_‐H_2_O, C_6_N_2_H_14_‐NH_4_Cl_3_, (C_4_N_2_H_12_)(NH_4_Br_3_)‐H_2_O, (C_4_N_2_H_12_)(NH_4_Br_3_)‐H_2_O, hmta‐NH_4_Br_3_, (H_2_DABCO)(NH_4_)[BF_4_]_3_, (H_2_A)[NH_4_(ClO_4_)_3_] (where H_2_A^2+^ cations = 1‐hydroxy‐1,4‐diazabicyclo[2.2.2]octane‐1,4‐diium, piperazine‐1,4‐diium, 1‐methyl‐piperazine‐1,4‐diium, homopiperazine‐1,4‐diium, 1‐methyl‐1,4‐diazabicyclo‐[2.2.2]octane‐1,4‐diium), (H_2_DABCO)‐NH_3_OH^+^‐(ClO_4_)_3_, and (H_2_DABCO)‐NH_2_NH_3_^+^‐(ClO_4_)_3_ have been successfully synthesized to show their potential in ferroelectric and piezoelectric applications.^[^ ^31^, ^34^, ^78^, ^79^, ^80^, ^81^ ^]^ A breakthrough on metal‐free organic perovskite ferroelectrics has been reported to include 23 new compounds with a general formula of A(NH_4_)X_3_ (A = divalent organic cation and X = Cl, Br, or I). Of these, MDABCO‐NH_4_I_3_ has been reported to show a spontaneous polarization of 22 mC cm^−2^ (close to that of BTO), a high phase transition temperature of 448 K (higher than BTO), and 8 obvious polarization directions.^[^ ^33^ ^]^

**Figure 4:**
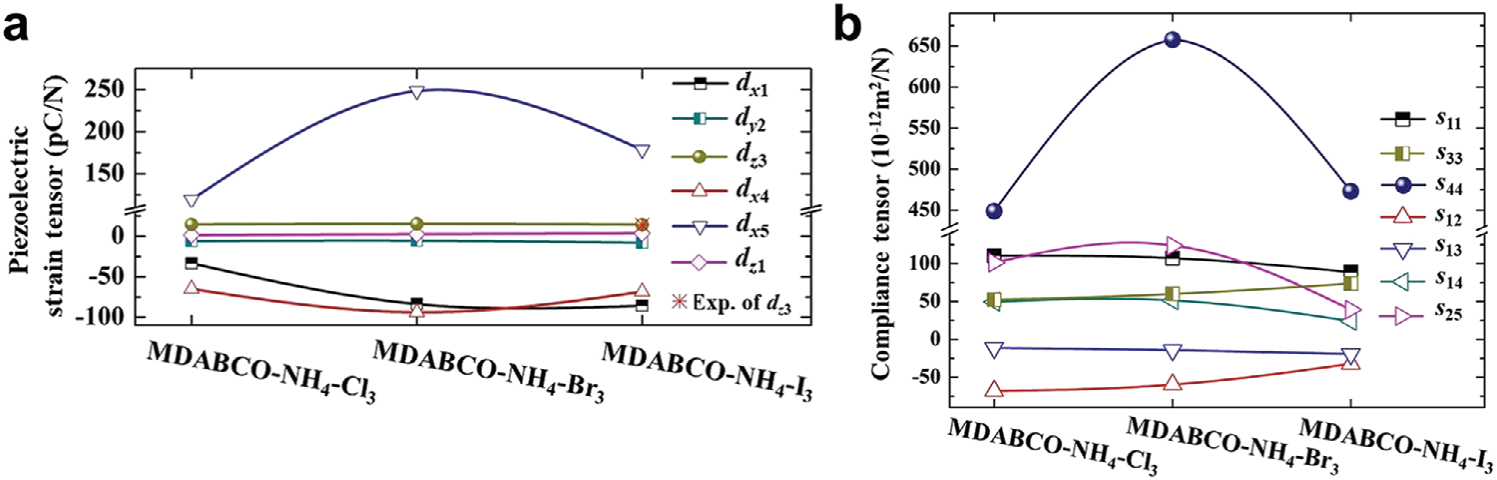
a) The calculated piezoelectric strain tensor of MDABCO‐NH_4_X_3_. b) The calculated elastic compliance tensor of MDABCO‐NH_4_X_3._ Reproduced under terms of Creative Commons Attribution 4.0 International license.^[^ ^57^ ^]^ Copyright 2019, The Authors, published by Springer Nature.

Further, the piezoelectric constant (d_33_) of organic MDABCO‐NH_4_I_3_ has been reported as 14 pC n ^−1^, which is lower than many competing materials (**Table** **1**). However, simulation results show that MDABCO‐NH_4_X_3_ type metal‐free perovskites exhibit large piezoelectric strain components in the d*_x_*_5_ direction (**Figure** **4**a), which can be originated from the large elastic compliance (Figure 4b).^[^ ^57^ ^]^ It reveals that MDABCO‐NH_4_X_3_ type metal‐free organic perovskites are good candidates for various piezoelectric applications as pressure sensors, actuators, generators, and piezotronic devices.

**Table 1:** Piezoelectric constants of various materials

Material	Piezoelectric constant [pC n ^−1^)	Reference
MDABCO‐NH_4_I_3_	d_33_ = 14	^[^ ^36^ ^]^
CH_3_NH_3_PbI_3_	d_33_ = 5.12	^[^ ^85^ ^]^
PVDF	d_33_ = 34	^[^ ^86^ ^]^
Poly‐L‐lactic acid (PLLA)	d_14_ = 11	^[^ ^87^ ^]^
Cellulose nanocrystal	d_25_ = 2.1	^[^ ^88^ ^]^
ZnO	d_33_ = 3	^[^ ^89^ ^]^
GaN	d_33_ = 3.1	^[^ ^90^ ^]^
PZT	d_33_ = 290	^[^ ^11^ ^]^
BaTiO_3_	d_33_ = 190	^[^ ^91^ ^]^
NaNbO_3_	d_33_ = 52.5	^[^ ^92^ ^]^
Diisopropylammonium bromide (DIPAB)	d_33_ = 11	^[^ ^93^ ^]^
LiNbO_3_	d_33_ = 11	^[^ ^28^ ^]^
ImClO_4_	d_33_ = 41	^[^ ^94^ ^]^
Croconic acid	d_33_ = 5	^[^ ^95^ ^]^
Triglycine sulfate	d_33_ = 22	^[^ ^28^ ^]^
Nylon	d_33_ = 2	^[^ ^28^ ^]^
(4‐aminotetrahydropyran)_2_PbBr_4_	d_33_ = 76	^[^ ^96^ ^]^
[Me_3_NCH_2_ClMnCl_3_, (TMCM‐MnCl_3_)]	d_33_ = 185	^[^ ^28^ ^]^
TMCM‐CdCl_3_	d_33_ = 220–240	^[^ ^28^ ^]^
Na_0.47_Bi_0.47_Ba_0.06_Ti_1−_ *_x_* Fe*_x_* O_3−Δ_ (BNBT‐100*x*Fe, *x* = 0.01)	d_33_ = 168	^[^ ^97^ ^]^
Ba(Ti_0.8_Zr_0.2_)O_3_(Ba_0.7_Ca_0.3_)TiO_3_	d_33_ ≈ 620	^[^ ^98^ ^]^
(Ba_0.94_Ca_0.06_)(Ti_0.95_Zr_0.05_)O_3_	d_33_ = 755	^[^ ^99^ ^]^
CuO‐doped (Ba,Ca)(Ti,Sn)O_3_	d_33_ = 683	^[^ ^100^ ^]^
Bi_0.5_Na_0.5_Ti_1−_ *_x_* Mn*_x_* O_3−*δ*_ (BNTM10000*x*, *x* = 0.25%)	d_33_ = 105	^[^ ^101^ ^]^
Zr‐modified Bi_0.5_(Na_0.78_K_0.22_)_0.5_TiO_3_ ceramics (BNKTZ‐100*_x_*, with *x* = 0)	d_33_ = 168	^[^ ^102^ ^]^
Bi_0.5_(Na_0.82_K_0.18_)_0.5_TiO_3_—*x*CuO (*x* = 0)	d_33_ = 146	^[^ ^103^ ^]^
0.93(Bi_0.5_Na_0.5_)TiO_3_‐0.07BaTiO_3_‐*x*Pr (BNBT‐*x*Pr, *x* = 0.003)	d_33_ = 194	^[^ ^26^ ^]^
[(Bi_1/2_Na_1/2_)_0.95_Ba_0.05_]_1−_ *_x_* La*_x_* TiO_3_ (*x* = 0.02)	d_33_ = 151	^[^ ^104^ ^]^
(1 − *x*)[0.67Bi_1.05_‐FeO_3_‐0.33BaTiO_3_]‐*x*Bi_1.05_(Zn_0.5_Ti_0.5_)O_3_ (*x* = 0.03)	d_33_ = 324	^[^ ^105^ ^]^
0.67Bi_1.05_‐(Fe_1−_ *_x_* Ga*_x_*)O_3_‐0.33BaTiO_3_ (*x* = 0.03)	d_33_ = 402	^[^ ^105^ ^]^
MnO_2_‐doped (K_0.5_Na_0.5_)NbO_3_	d_33_ = 270	^[^ ^106^ ^]^
K_0.8_Na_0.2_NbO_3_	d_33_ = 110	^[^ ^107^ ^]^
K_0.47_Na_0.53_NbO_3_	d_33_ = 220	^[^ ^108^ ^]^
Rhombohedral 0.72 Pb(Mg_1/3_Nb_2/3_)O_3_ – 0.28 PbTiO_3_ (PMN‐28PT)	d_33_ > 1200	^[^ ^109^ ^]^

Moreover, as shown in Table 1, the huge differences in piezoelectric constants of metal‐halide perovskites and metal‐free perovskites can be attributed to several structural factors, such as phase transition, dependence on polar direction, and elemental composition. Typically, the piezoelectric coefficient can be expressed by Equation (1) below:

(1) $$d_{33}=2Q_{33\;}\;\varepsilon_{33}\;P_{r}$$

where *d*_33_ is the piezoelectric coefficient, 2*Q*_33 _ is the electrostriction constant, *ε*_33_ is the permittivity, and *P*_r_ is the remanent polarization.^[^ ^82^ ^]^ The above formula could be further written in tensor form where piezoelectric constants of different directions are involved. As shown in Equation (2) below:

(2) $$\begin{array}{ccc} d_{mij} & = & \frac{\partial S_{ij}}{\partial E_{m}}=\frac{Q_{ijkl}P_{k}\partial P_{l}}{\partial E_{m}}+\frac{Q_{ijkl}P_{l}\partial P_{k}}{\partial E_{m}}\\ & = & Q_{ijkl}\;P_{k}\varepsilon_{lm}+Q_{ijkl}P_{l}\varepsilon_{km} \end{array}$$

*d_mij_* is the piezoelectric coefficient, *S_ij_* is the strain, *E_m_* is the electric field, *Q_ijkl_* is the electrostriction coefficient, *P_k_* and *P_l_* are the polarizations, and *ε*_*km*_ and *ε*_*lm*_ are the dielectric permittivities.^[^ ^83^ ^]^

Piezoelectric materials possessing their own polar axis or polar plane will lead to the diverse piezoelectric coefficient in each direction. For example, MDABCO‐NH_4_I_3_ has been reported to exhibit the highest piezoelectric constant of d_15_ = 179 pm V^−1^ due to its preferred orientation along <111>.^[^ ^57^, ^84^ ^]^ Additionally, the electromechanical coupling induced by MPB is another reason that certain piezoelectric materials possess remarkable values in d_33_ (Table 1). However, this mostly occurs in materials with solid solution instead of pure elements.

### 3.1 Piezoelectric Sensors

**Figure 5:**
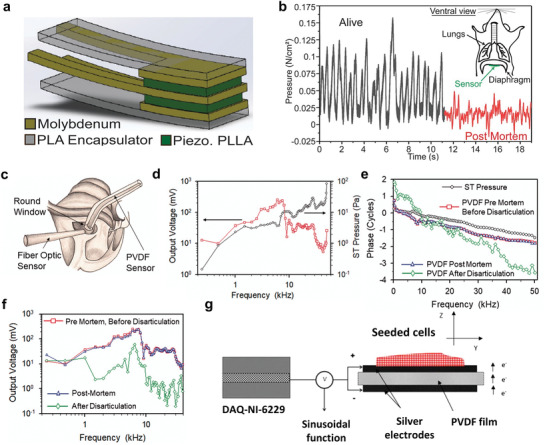
a) Schematic of a biodegradable piezoelectric Poly‐L‐lactic acid (PLLA) sensor. b) Distinct force signal produced by PLLA sensor when the mouse was alive and under anesthesia (black), and when the mouse was euthanized by an overdose of anesthetics (red). Reproduced with permission.^[^ ^87^ ^]^ Copyright 2018, National Academy of Sciences. c) Schematic of fiber optic and PVDF pressure sensors inserted into the round window of a gerbil cochlea. d) Output voltage measured with PVDF sensor (red) and pressure in the scala tympani measured with fiber optic sensor (black). e) Phase measured with fiber optic pressure sensor (black circle), PVDF sensor pre‐mortem and before disarticulation (red squares), postmortem (blue triangles), and after disarticulation (greed diamonds). f) Output voltage measured with PVDF sensor pre‐mortem or before disarticulation (red squares), postmortem (blue triangles), and after disarticulation (green diamonds). Reproduced under terms of Creative Commons Attribution 4.0 International license.^[^ ^116^ ^]^ Copyright 2018, The Authors, published by SAGE. g) Illustration of PVDF‐based actuators and tissue stimulators of the cell culture exposed to micro‐vibration. Reproduced with permission.^[^ ^126^ ^]^ Copyright 2010, Elsevier.

Piezoelectric sensors for monitoring physiological signals have become a popular research field due to advancements in wearable electronics technology and the development of Internet of Things (IoT).^[^ ^110^ ^]^ Notably, lead‐free materials like BaTiO_3_, ZnO, GaN, and ZnSnO_3_ have become popular candidates owing to their excellent environmental friendliness, sustainability, and biocompatibility.^[^ ^111^, ^112^, ^113^, ^114^ ^]^ For example, the fabrication of a ZnO and ZnSnO_3_ nanowire‐based piezoelectric strain sensors have been designed to exhibit high flexibility and gauge factors (the ratio of relative change in electrical resistance to the mechanical strain) of 1250 and 3740, respectively.^[^ ^112^, ^114^ ^]^ This can be attributed to the higher polarization of ZnSnO_3_ (59 µC cm^−2^) as compared to ZnO (≈5 µC cm^−2^), indicating that the piezoelectric potential induced by polarization will affect the sensitivity of piezoelectric sensors. On the other hand, metal‐free perovskites, such as MDABCO‐NH_4_I_3_ which exhibit higher resistivity (i.e., lower mobility and larger bandgap) and strong spontaneous polarization can also be a potential candidate for piezoelectric sensor applications. It has been shown that organic piezoelectric films were explored as sensor applications due to a low‐cost and low‐temperature processing requirement. The sensors fabricated using a compressive bandage can be useful and their biodegradability is desired for hygiene purposes.^[^ ^115^ ^]^ In addition to in vitro applications, in vivo applications of using organic films for piezoelectric pressure sensors (**Figure** **5**a,b) and cochlear implants (Figure 5c–f) have also been developed.^[^ ^87^, ^116^ ^]^ Although the biodegradability of metal‐free perovskites has yet to be studied, the synergic properties of metal‐free and organic molecular structure are highly desired for achieving the above‐mentioned demands.

### 3.2 Piezoelectric Actuators

The working principle of the actuator is based on the inverse piezoelectric behavior and electrostrictive response induced by Maxwell stress or intrinsic electrostrictive effect.^[^ ^83^ ^]^ The inverse piezoelectric effect is a first‐order electro‐mechanical coupling in which strain and electric field show linear relation. As an electric field is applied to a piezoelectric material, localized dipole moments are generated due to the stretching of the material along the direction of the electric field.^[^ ^83^ ^]^ These mechanisms have been widely applied in ultrasonics, sonars, position controllers, and microelectromechanical systems (MEMs).^[^ ^117^, ^118^ ^]^ The electrostrictive response is a second‐order electro‐mechanical coupling in which the strain is proportional to the square of the electric field. The intrinsic electrostrictive response is generated by a harmonic displacement of positive ions and negative ions under applied bias. Maxwell stress originates from the imperfect screening on electrodes where the uncompensated charges form Coulombic interactions and induce the strain.^[^ ^83^, ^119^ ^]^

To the best of our knowledge, only few literatures have addressed the electrostriction constant (Q) of metal‐free perovskites and their corresponding actuator performance. Nevertheless, it may be inferred from the piezoelectric formula in the tensor form (Equation (2)). The relationship between the piezoelectric coefficient and the electrostrictive coefficient expressed by tensor calculation indicates that electrostrictive coefficient is proportional to piezoelectric coefficient.^[^ ^83^ ^]^ For example, if a specific perovskite possesses high d_15_, it also possesses high Q_15_. Further, the correlation between electric field and polarization is given by Equation (3) below:

(3) $$S_{ij=}\;Q_{ijkl}P_{k}\;P_{l}=M_{ijkl}\;E_{k}E_{l}$$

where *S_ij_* is the strain, *Q_ijkl_* and *M_ijkl_* are electrostriction coefficients, *P_k_* and *P_l_* are polarizations, and *E_k_* and *E_l_* are electric fields. Herein, the electric field‐induced strain is proportional to the square of polarization. Therefore, the metal‐free perovskites with high polarization might exhibit good performance in actuator applications.

Previously, a large electrostrictive coefficient (Q_33_) was observed in only relaxor‐based ferroelectric materials like Pb(Zn_1/3_Nb_2/3_)O_3_—PbTiO_3_, Pb(Mg_1/3_Nb_2/3_)O_3_—PbTiO_3_, Ba(Zr_0.2_Ti_0.8_)O_3−_ *_x_* (Ba_0.7_Ca_0.3_)TiO_3_, and PVDF composites.^[^ ^120^, ^121^, ^122^, ^123^, ^124^, ^125^ ^]^ The organic PVDF composites‐based actuator and tissue stimulator shown in Figure 5g consists of a thin film of PVDF printed with silver ink on both sides as electrodes.^[^ ^126^ ^]^ Recently, MAPbI_3_, an organic–inorganic metal halide perovskite has been reported to exhibit a large electrostrictive response (Q_33_ = −730 nm^2^ V^−2^) originated from lattice deformation caused by iodine Frenkel defects.^[^ ^119^ ^]^ The results suggests that halide‐based perovskites might hold a great potential in electrostriction applications due to their low defect formation energy and high defect tolerance.^[^ ^127^, ^128^ ^]^ In addition, it has been demonstrated that chirality can induce relaxor properties and bring out striking electrostriction behaviors.^[^ ^125^ ^]^ The combination of defect‐induced deformation and chirality induced electrostriction in metal‐free perovskites can provide diverse potential applications in non‐toxic biomedical MEMs actuators.

### 3.3 Piezoelectric Generators

**Figure 6:**
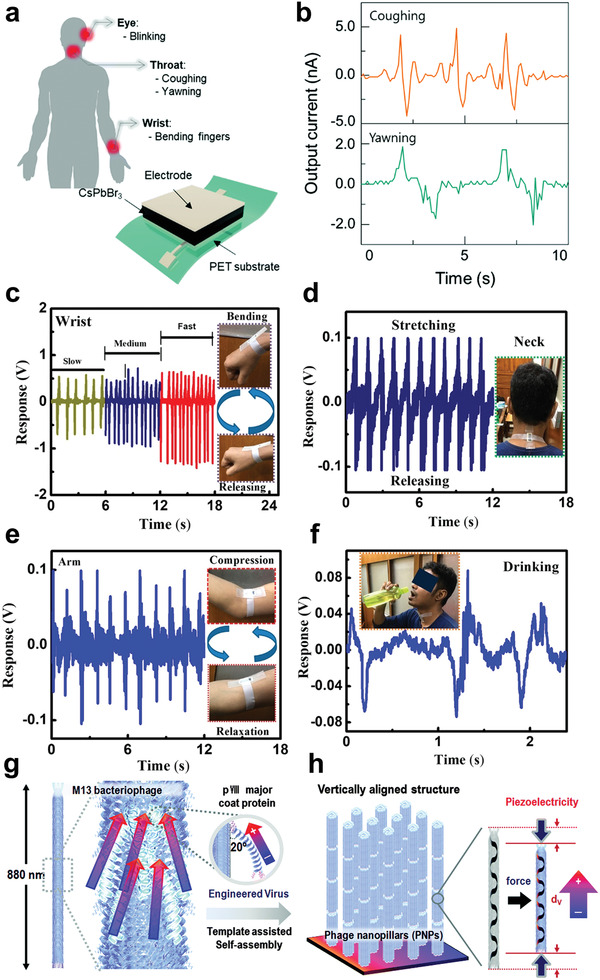
a) Device schematic of a CsPbBr_3_ nanogenerator and self‐powered physiological signal characterizations from b) coughing/yawning. Reproduced with permission.^[^ ^129^ ^]^ Copyright 2020, Royal Society of Chemistry. Output of the real‐time sensor in terms of waveforms measured from c) wrist, d) neck e) arm, and f) drinking movements. Reproduced with permission.^[^ ^130^ ^]^ Copyright 2020, American Chemical Society. Schematics of g) aligned M13 bacteriophage nanopillars and h) the piezoelectric dipole on vertical direction. Reproduced with permission.^[^ ^21^ ^]^ Copyright 2015, Royal Society of Chemistry.

**Figure 7:**
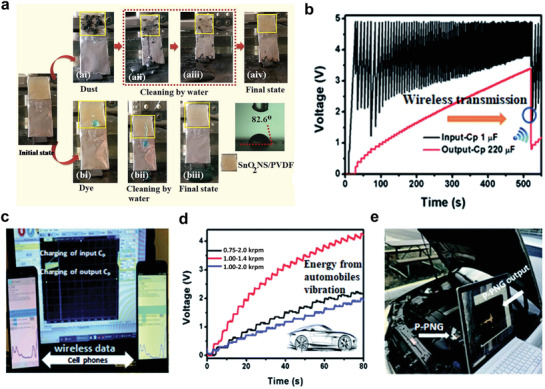
a) A SnO_2_/PVDF PENG with self‐cleaning ability. Reproduced with permission.^[^ ^134^ ^]^ Copyright 2019, Elsevier. Schematic representation of b) self‐powered integrated wireless electronic node (SIWEN) applications in hybrid perovskite‐based nanocomposites and c) signal transmission by cellphones. d) Charging a 1 µF capacitor by a single perovskite/polymer PENG while exciting by an automobile engine and e) engine vibration detection scene. Reproduced with permission.^[^ ^138^ ^]^ Copyright 2020, Royal Society of Chemistry.

The power shortage problem has been treated as a critical issue for current wearable electronics. Therefore, auxiliary power technologies have been proposed to solve these issues, and piezoelectric generator (PENG) is one of the typical examples. Perovskite‐based PENGs have been widely investigated in various technological aspects owing to their outstanding piezoelectric properties.^[^ ^129^ ^]^ The origin of the ultra‐high piezoelectric output of inorganic halide perovskite has been discovered as shown in **Figure** **6**a,b.^[^ ^129^ ^]^ Due to the high piezoelectric constant (d_33_) of *β*‐phase PVDF, it has also been widely used as the thin film PENG or the matrix of piezoelectric nanocomposites.^[^ ^86^ ^]^ For example, a self‐powered human health monitoring e‐skin sensor using highly aligned PVDF nanofibers arrays has also been developed and shown in Figure 6c–f.^[^ ^130^ ^]^ Other organic piezoelectric materials such as PLA (polylactic acid), chitin, chitosan, cellulose, and even M13 bacteriophage have shown potential as piezoelectric nanogenerators.^[^ ^21^, ^105^, ^131^, ^132^, ^133^ ^]^ The characteristics of bacteriophage piezoelectric nanogenerators are indicated in Figure 6g,h. Recently, SnO_2_ sheet/PVDF nanocomposites with a self‐cleaning ability for smart wearable energy harvesters or sensors have also been fabricated and shown in **Figure** **7**a.^[^ ^134^ ^]^

Due to the unique properties of lead‐based halide perovskites, several attempts have been made in exploring related applications, such as pyroelectric, flexoelectric, triboelectric, and piezoelectric.^[^ ^85^, ^135^, ^136^, ^137^ ^]^ For instance, MAPbI_3_ has been demonstrated to be applicable for PENG design.^[^ ^85^ ^]^ Additionally, FAPbBr_2_I/PVDF nanocomposites designed as self‐powered integrated wireless electronic node (SIWEN) devices are shown in Figure 7b–e.^[^ ^138^ ^]^ On the other hand, metal‐free perovskites can also possibly be explored due to non‐toxicity. According to the simulation results, MDABCO‐NH_4_X_3_ series have large piezoelectric responses in the d*_x_*_5_ direction, showing great potential in high power density PENGs to be integrated in Internet of Things (IoT) devices or physiological monitoring systems.^[^ ^51^ ^]^

### 3.4 From Piezoelectronics to Piezotronics

**Figure 8:**
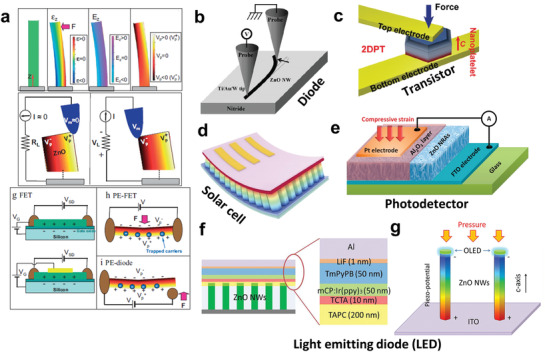
a) Principles of piezotronics in a nanowire (up) and a field‐effect transistor (bottom). Reproduced with permission.^[^ ^139^ ^]^ Copyright 2007, Wiley‐VCH. b) Strain‐gated ZnO nanowire Schottky diode. Reproduced with permission.^[^ ^140^ ^]^ Copyright 2007, Wiley‐VCH. c) Strain‐gated field‐effect transistor. Reproduced with permission.^[^ ^141^ ^]^ Copyright 2017, Wiley‐VCH. d) Piezotronic effect‐enhanced perovskite solar cell. Reproduced with permission.^[^ ^143^ ^]^ Copyright 2019, American Chemical Society. e) Metal‐insulator‐semiconductor structured photodetector based on ZnO nanorods. Reproduced with permission.^[^ ^142^ ^]^ Copyright 2014, Elsevier. f) Piezotronic effect‐enhanced organic light‐emitting diode. g) Piezopotential distribution in a ZnO nanowire and enhanced current and light emission of the device. Reproduced with permission.^[^ ^144^ ^]^ Copyright 2017, American Chemical Society.

The word “piezotronics” is the combination of “piezoelectrics” and “electronics” that first proposed in 2007 by coupling piezoelectric and semiconducting properties of materials.^[^ ^139^ ^]^ Piezotronics can be applied to design and fabricate electronic devices, such as piezoelectric‐gated diodes, piezoelectric‐gated transistors, and piezotronic effect‐enhanced photodetectors, solar cells, and light‐emitting diodes shown in **Figure** **8**a–g.^[^ ^139^, ^140^, ^141^, ^142^, ^143^, ^144^ ^]^ Besides, it can also be widely used in sensors, energy harvesters, human‐robotic interfaces, and self‐powered devices. For halide perovskite piezotronics, examples including piezotronic‐enhanced solar cells and photodetectors have been reported.^[^ ^143^, ^145^ ^]^ It is expected that metal‐free perovskites may also contribute to piezotronics devices such as piezotronic enhanced X‐ray photodetector or strain gated transistors.^[^ ^145^ ^]^ However, piezotronics devices based on metal‐free perovskites are still scarce due to their low mobilities and large bandgaps. The highest occupied molecular orbital (HOMO) and lowest unoccupied molecular orbital (LUMO) of metal‐free perovskites are defined by the A‐site organics.^[^ ^64^ ^]^ Therefore, by properly designed A‐site cations, it is possible to overcome the barrier of low mobility (DABCO‐NH_4_Br_3_: 2.08 cm^2^V^−1^s^−1^) and large bandgap (MDABCO‐NH_4_I_3_: 3.9 eV).^[^ ^33^, ^145^ ^]^ This can largely enhance the potential of metal‐free perovskites in either piezotronics or semiconductor applications.

## 4 Challenges and Outlooks

Although metal‐free perovskites have been reported to have outstanding performance in various applications, the stability, durability, and toxicity are still critical issues that remain to be solved. In terms of stability, despite their superior advantages in eco‐friendliness and sustainability (compared to lead‐halide perovskites or inorganic semiconductors), the recent developments on metal‐free perovskites emerge as a crucial challenge on the material and device instability and thereby limit large‐scale production as well as commercial feasibility. Substituting a stable metal cation with organic cation apparently diminishes the crystal's bonding network. Such metal‐free perovskites mainly rely on the hydrogen bonding during the formation of ABX_3_ crystal, the thermodynamic and environmental stability (i.e., oxygen, moisture, UV light, heat, and chemicals exposure) need to be thoroughly considered and further tested upon the development of highly stable metal‐free perovskites.^[^ ^60^, ^64^ ^]^ The most efficient way to enhance the stability and durability of halide perovskites is to encapsulate them with hydrophobic polymer or to develop halide perovskite/polymer composites. Therefore, halide perovskite composites based on biodegradable matrix‐like cellulose and PVA have been recently investigated to exhibit strong improvement on stability. Moreover, tuning metal‐free perovskites by A‐site or X‐site substitution has been demonstrated as an effective approach to show distinct properties. For example, MPB can be utilized to enhance the electro‐mechanical properties by inducing the polarization rotation. Furthermore, due to the widely developed techniques for synthesizing halide perovskite nanostructures, engineering metal‐free perovskite nanostructures based on nanocrystals, nanoplatelets, nanoarrays, and nanowires can be considered the next step. For instance, halide perovskites nanoarrays for piezoelectric energy harvesting and miniature photovoltaic devices have been successfully demonstrated. Therefore, it is expected to utilize metal‐free perovskites for various applications via effective structure engineering approaches.

## 5 Conclusion

In this perspective, we have summarized the fundamental mechanisms and recently designed metal halide perovskite‐based piezoelectric devices that can possibly be implemented for various metal‐free perovskites. The design theories of ABX_3_ perovskite structures in terms of symmetry breaking have also been discussed. The factors which induce or enhance the electromechanical property of the organic perovskite such as external stimulus and MPB have also been discussed. Various possible applications of metal‐free material‐based piezoelectric devices such as piezoelectric sensors, actuators, nanogenerators, piezotronic devices were explained. In the last, challenges and outlooks of piezoelectric metal‐free organic perovskites for various aspects were highlighted for future applications.

## Conflict of Interest

The authors declare no conflict of interest.

## References

[1] G. Rose , Ann. Phys. 1839, 124, 551.

[2] J. F. Scott , Science 2007, 315, 954. 17303745 10.1126/science.1129564

[3] H. Takasu , J. Electroceram. 2000, 4, 327.

[4] P. Tonui , S. O. Oseni , G. Sharma , Q. Yan , G. T. Mola , Renewable Sustainable Energy Rev. 2018, 91, 1025.

[5] S. F. Hoefler , G. Trimmel , T. Rath , Monatsh. Chem. ‐ Chem. Mon. 2017, 148, 795. 10.1007/s00706-017-1933-9 PMC5387038 28458399

[6] Z. Zhu , Q. Sun , Z. Zhang , J. Dai , G. Xing , S. Li , X. Huang , W. Huang , J. Mater. Chem. C 2018, 6, 10121.

[7] W. Zhang , R. G. Xiong , Chem. Rev. 2012, 112, 1163. 21939288 10.1021/cr200174w

[8] J. Seidel , D. Fu , S. Y. Yang , E. Alarcón‐Lladó , J. Wu , R. Ramesh , J. W. Ager III , Phys. Rev. Lett. 2011, 107, 126805. 22026787 10.1103/PhysRevLett.107.126805

[9] Z. X. Zhang , H. Y. Zhang , W. Zhang , X. G. Chen , H. Wang , R. G. Xiong , J. Am. Chem. Soc. 2020, 142, 17787. 33002358 10.1021/jacs.0c09288

[10] Y. Zhang , D. Sun , J. Gao , X. Hua , X. Chen , G. Mei , W. Liao , Chem. ‐ Asian J. 2019, 14, 1028. 30756525 10.1002/asia.201801921

[11] B. Jaffe , J. Am. Ceram. Soc. 1958, 41, 494.

[12] M. T. Chorsi , E. J. Curry , H. T. Chorsi , R. Das , J. Baroody , P. K. Purohit , H. Ilies , T. D. Nguyen , Adv. Mater. 2019, 31, 1802084. 10.1002/adma.201802084 30294947

[13] K. Wang , F. Yao , W. Jo , D. Gobeljic , V. V. Shvartsman , D. C. Lupascu , J. Li , J. Rödel , Adv. Funct. Mater. 2013, 23, 4079.

[14] L. Persano , C. Dagdeviren , Y. Su , Y. Zhang , S. Girardo , D. Pisignano , Y. Huang , J. A. Rogers , Nat. Commun. 2013, 4, 1633. 23535654 10.1038/ncomms2639

[15] Z. Li , G. Zhu , R. Yang , A. C. Wang , Z. L. Wang , Adv. Mater. 2010, 22, 2534. 20446305 10.1002/adma.200904355

[16] J. Gao , X. Ke , M. Acosta , J. Glaum , X. Ren , MRS Bull. 2018, 43, 595.

[17] K. Park , J. H. Son , G. Hwang , C. K. Jeong , J. Ryu , M. Koo , I. Choi , S. H. Lee , M. Byun , Z. L. Wang , Adv. Mater. 2014, 26, 2514. 24523251 10.1002/adma.201305659

[18] K. Y. Lee , M. K. Gupta , S. W. Kim , Nano Energy 2015, 14, 139.

[19] M. El Achaby , F. Z. Arrakhiz , S. Vaudreuil , E. M. Essassi , A. Qaiss , Appl. Surf. Sci. 2012, 258, 7668.

[20] A. Kholkin , N. Amdursky , I. Bdikin , E. Gazit , G. Rosenman , ACS Nano 2010, 4, 610. 20131852 10.1021/nn901327v

[21] D. M. Shin , H. J. Han , W. G. Kim , E. Kim , C. Kim , S. W. Hong , H. K. Kim , J. W. Oh , Y. H. Hwang , Energy Environ. Sci. 2015, 8, 3198.

[22] D. Kim , S. A. Han , J. H. Kim , J. Lee , S. Kim , S. Lee , Adv. Mater. 2020, 32, 1906989. 10.1002/adma.201906989 32103565

[23] Y. R. Zhang , J. F. Li , B. P. Zhang , C. E. Peng , J. Appl. Phys. 2008, 103, 074109.

[24] M. H. Zhang , K. Wang , Y. J. Du , G. Dai , W. Sun , G. Li , D. Hu , H. C. Thong , C. Zhao , X. Q. Xi , J. Am. Chem. Soc. 2017, 139, 3889. 28233999 10.1021/jacs.7b00520

[25] X. Wang , J. Wu , D. Xiao , J. Zhu , X. Cheng , T. Zheng , B. Zhang , X. Lou , X. Wang , J. Am. Chem. Soc. 2014, 136, 2905. 24499419 10.1021/ja500076h

[26] Q. Yao , F. Wang , F. Xu , C. M. Leung , T. Wang , Y. Tang , X. Ye , Y. Xie , D. Sun , W. Shi , ACS Appl. Mater. Interfaces 2015, 7, 5066. 25664585 10.1021/acsami.5b00420

[27] V. Basavalingappa , S. Bera , B. Xue , J. O'Donnell , S. Guerin , P. A. Cazade , H. Yuan , E. ul Haq , C. Silien , K. Tao , L. J. W. Shimon , S. A. M. Tofail , D. Thompson , S. Kolusheva , R. Yang , Y. Cao , E. Gazit , ACS Nano 2020, 14, 7025. 32441511 10.1021/acsnano.0c01654 PMC7315635

[28] Y. M. You , W. Q. Liao , D. Zhao , H. Y. Ye , Y. Zhang , Q. Zhou , X. Niu , J. Wang , P. F. Li , D. W. Fu , Z. Wang , S. Gao , K. Yang , J. M. Liu , J. Li , Y. Yan , R. G. Xiong , Science 2017, 357, 306. 28729511 10.1126/science.aai8535

[29] W. Q. Liao , D. Zhao , Y. Y. Tang , Y. Zhang , P. F. Li , P. P. Shi , X. G. Chen , Y. M. You , R. G. Xiong , Science 2019, 363, 1206. 30872522 10.1126/science.aav3057

[30] J. Harada , N. Yoneyama , S. Yokokura , Y. Takahashi , A. Miura , N. Kitamura , T. Inabe , J. Am. Chem. Soc. 2018, 140, 346. 29224333 10.1021/jacs.7b10539

[31] C. A. Bremner , M. Simpson , W. T. A. Harrison , J. Am. Chem. Soc. 2002, 124, 10960. 12224926 10.1021/ja027484e

[32] H. Y. Zhang , Y. Y. Tang , P. P. Shi , R. G. Xiong , Acc. Chem. Res. 2019, 52, 1928. 30986035 10.1021/acs.accounts.8b00677

[33] H. Y. Ye , Y. Y. Tang , P. F. Li , W. Q. Liao , J. X. Gao , X. N. Hua , H. Cai , P. P. Shi , Y. M. You , R. G. Xiong , Science 2018, 361, 151. 30002249 10.1126/science.aas9330

[34] H. Morita , R. Tsunashima , S. Nishihara , K. Inoue , Y. Omura , Y. Suzuki , J. Kawamata , N. Hoshino , T. Akutagawa , Angew. Chem., Int. Ed. 2019, 58, 9184. 10.1002/anie.201905087 31070833

[35] W. Y. Zhang , Y. Y. Tang , P. F. Li , P. P. Shi , W. Q. Liao , D. W. Fu , H. Y. Ye , Y. Zhang , R. G. Xiong , J. Am. Chem. Soc. 2017, 139, 10897. 28719192 10.1021/jacs.7b06013

[36] J. Young , J. M. Rondinelli , Phys. Rev. Mater. 2018, 2, 065406.

[37] G. Gou , J. Young , X. Liu , J. M. Rondinelli , Inorg. Chem. 2017, 56, 26. 27682844 10.1021/acs.inorgchem.6b01701

[38] Z. X. Chen , Y. Chen , Y. S. Jiang , J. Phys. Chem. B 2002, 106, 9986.

[39] H. Y. Zhang , Z. X. Zhang , X. G. Chen , X. J. Song , Y. Zhang , R. G. Xiong , J. Am. Chem. Soc. 2021, 143, 1664. 33449687 10.1021/jacs.0c12907

[40] P. Curie , J. Phys. Theor. Appl. 1894, 3, 393.

[41] Y. Y. Tang , P. F. Li , W. Q. Liao , P. P. Shi , Y. M. You , R. G. Xiong , J. Am. Chem. Soc. 2018, 140, 8051. 29894637 10.1021/jacs.8b04600

[42] D. K. Bharti , M. K. Gupta , R. Kumar , N. Sathish , A. K. Srivastava , Nano Energy 2020, 73, 104821.

[43] G. Zhu , Z. Zeng , L. Zhang , X. Yan , Comput. Mater. Sci. 2008, 44, 224.

[44] S. S. Chauhan , U. M. Bhatt , P. Gautam , S. Thote , M. M. Joglekar , S. K. Manhas , Sens. Actuators, A 2020, 304, 111879.

[45] D. B. Kim , K. H. Park , Y. S. Cho , Energy Environ. Sci. 2020, 13, 2077.

[46] M. Coll , A. Gomez , E. Mas‐Marza , O. Almora , G. Garcia‐Belmonte , M. Campoy‐Quiles , J. Bisquert , J. Phys. Chem. Lett. 2015, 6, 1408. 26263143 10.1021/acs.jpclett.5b00502

[47] L. L. Chu , T. Zhang , W. Y. Zhang , P. P. Shi , J. X. Gao , Q. Ye , D. W. Fu , J. Phys. Chem. Lett. 2020, 11, 1668. 32040321 10.1021/acs.jpclett.9b03556

[48] J. Wu , D. Xiao , J. Zhu , Chem. Rev. 2015, 115, 2559. 25792114 10.1021/cr5006809

[49] M. Ahart , M. Somayazulu , R. E. Cohen , P. Ganesh , P. Dera , H. Mao , R. J. Hemley , Y. Ren , P. Liermann , Z. Wu , Nature 2008, 451, 545. 18235495 10.1038/nature06459

[50] H. Fu , R. E. Cohen , Nature 2000, 403, 281. 10659840 10.1038/35002022

[51] D. Damjanovic , Appl. Phys. Lett. 2010, 97, 062906.

[52] C. J. Bartel , C. Sutton , B. R. Goldsmith , R. Ouyang , C. B. Musgrave , L. M. Ghiringhelli , M. Scheffler , Sci. Adv. 2019, 5, eaav0693. 30783625 10.1126/sciadv.aav0693 PMC6368436

[53] W. Q. Liao , D. Zhao , Y. Y. Tang , Y. Zhang , P. F. Li , P. P. Shi , X. G. Chen , Y. M. You , R. G. Xiong , Science 2019, 363, 1206. 30872522 10.1126/science.aav3057

[54] Y. Xie , B. X. Wang , N. Claire , Z. G. Ye , J. Am. Ceram. Soc. 2022, 105, 1450.

[55] D. Xue , P. V. Balachandran , H. Wu , R. Yuan , Y. Zhou , X. Ding , J. Sun , T. Lookman , Appl. Phys. Lett. 2017, 111, 032907.

[56] R. Wang , K. Wang , F. Yao , J. F. Li , F. H. Schader , K. G. Webber , W. Jo , J. Rödel , J. Am. Ceram. Soc. 2015, 98, 2177.

[57] H. Wang , H. Liu , Z. Zhang , Z. Liu , Z. Lv , T. Li , W. Ju , H. Li , X. Cai , H. Han , npj Comput. Mater. 2019, 5, 17.

[58] Z. Shi , J. Guo , Y. Chen , Q. Li , Y. Pan , H. Zhang , Y. Xia , W. Huang , Adv. Mater. 2017, 29, 1605005. 10.1002/adma.201605005 28160346

[59] Z. Xiao , Z. Song , Y. Yan , Adv. Mater. 2019, 31, 1803792. 10.1002/adma.201803792 30680809

[60] F. Zhang , Z. Ma , Z. Shi , X. Chen , D. Wu , X. Li , C. Shan , Energy Mater. Adv. 2021, 2021, 5198145.

[61] R. E. Brandt , V. Stevanović , D. S. Ginley , T. Buonassisi , MRS Commun. 2015, 5, 265.

[62] M. Shimizu , M. Koshimizu , Y. Fujimoto , T. Yanagida , S. Ono , K. Asai , Opt. Mater. 2016, 61, 115.

[63] H. Hu , B. Dong , W. Zhang , J. Mater. Chem. A 2017, 5, 11436.

[64] X. Song , G. Hodes , K. Zhao , S. Liu , Adv. Energy Mater. 2021, 11, 2003331.

[65] W. Ke , C. C. Stoumpos , M. Zhu , L. Mao , I. Spanopoulos , J. Liu , O. Y. Kontsevoi , M. Chen , D. Sarma , Y. Zhang , M. R. Wasielewski , M. G. Kanatzidis , Sci. Adv. 2017, 3, e1701293. 28875173 10.1126/sciadv.1701293 PMC5576879

[66] M. V. Kovalenko , L. Protesescu , M. I. Bodnarchuk , Science 2017, 358, 745. 29123061 10.1126/science.aam7093

[67] J. Bie , D.‐B. Yang , M.‐G. Ju , Q. Pan , Y.‐M. You , W. Fa , X. C. Zeng , S. Chen , JACS Au 2021, 1, 475. 34467310 10.1021/jacsau.1c00014 PMC8395623

[68] N. Parikh , M. Karamta , N. Yadav , M. Mahdi Tavakoli , D. Prochowicz , S. Akin , A. Kalam , S. Satapathi , P. Yadav , J. Energy Chem. 2022, 66, 74.

[69] T. Nakajima , K. Sawada , J. Phys. Chem. Lett. 2017, 8, 4826. 28927268 10.1021/acs.jpclett.7b02203

[70] G. Pilania , A. Mannodi‐Kanakkithodi , B. P. Uberuaga , R. Ramprasad , J. E. Gubernatis , T. Lookman , Sci. Rep. 2016, 6, 19375. 26783247 10.1038/srep19375 PMC4726030

[71] S. Lu , Q. Zhou , Y. Ouyang , Y. Guo , Q. Li , J. Wang , Nat. Commun. 2018, 9, 3405. 30143621 10.1038/s41467-018-05761-w PMC6109147

[72] T. Wu , J. Wang , Nano Energy 2019, 66, 104070.

[73] R. Jacobs , G. Luo , D. Morgan , Adv. Funct. Mater. 2019, 29, 1804354.

[74] Z. Li , Q. Xu , Q. Sun , Z. Hou , W.‐J. Yin , Adv. Funct. Mater. 2019, 29, 1807280.

[75] R. Yuan , Z. Liu , P. V. Balachandran , D. Xue , Y. Zhou , X. Ding , J. Sun , D. Xue , T. Lookman , Nat. Commun. 2018, 30, 1702884.

[76] P. V. Balachandran , A. A. Emery , J. E. Gubernatis , T. Lookman , C. Wolverton , A. Zunger , Phys. Rev. Mater. 2018, 2, 043802.

[77] M. Saliba , Adv. Energy Mater. 2019, 9, 1803754.

[78] K. Li , L.‐Y. Dong , H.‐X. Xu , Y. Qin , Z.‐G. Li , M. Azeem , W. Li , X.‐H. Bu , Mater. Chem. Front. 2019, 3, 1678.

[79] L.‐L. Chu , T. Zhang , W.‐Y. Zhang , P.‐P. Shi , J.‐X. Gao , Q. Ye , D.‐W. Fu , J. Phys. Chem. Lett. 2020, 11, 1668. 32040321 10.1021/acs.jpclett.9b03556

[80] Y. Shang , R.‐K. Huang , S.‐L. Chen , C.‐T. He , Z.‐H. Yu , Z.‐M. Ye , W.‐X. Zhang , X.‐M. Chen , Cryst. Growth Des. 2020, 20, 1891.

[81] Y. Shang , Z.‐H. Yu , R.‐K. Huang , S.‐L. Chen , D.‐X. Liu , X.‐X. Chen , W.‐X. Zhang , X.‐M. Chen , Engineering 2020, 6, 1013.

[82] G. Helke , K. Lubitz , in Piezoelectricity: Evolution and Future of a Technology, Vol. 114 (Eds: W. Heywang , K. Lubitz , W. Wersing ), Springer Berlin Heidelberg, Berlin, Heidelberg 2008, p. 89.

[83] F. Li , L. Jin , Z. Xu , S. Zhang , Appl. Phys. Rev. 2014, 1, 011103.

[84] D. J. W. Allen , N. C. Bristowe , A. L. Goodwin , H. H. M. Yeung , J. Mater. Chem. C 2021, 9, 2706. 10.1039/d1tc00619c PMC8905487 35359799

[85] Y. J. Kim , T. V. Dang , H. J. Choi , B. J. Park , J. H. Eom , H. A. Song , D. Seol , Y. Kim , S. H. Shin , J. Nah , J. Mater. Chem. A 2016, 4, 756.

[86] J. Gomes , J. S. Nunes , V. Sencadas , S. Lanceros‐Méndez , Smart Mater. Struct. 2010, 19, 065010.

[87] E. J. Curry , K. Ke , M. T. Chorsi , K. S. Wrobel , A. N. Miller III , A. Patel , I. Kim , J. Feng , L. Yue , Q. Wu , C. L. Kuo , K. W. H. Lo , C. T. Laurencin , H. Ilies , P. K. Purohit , T. D. Nguyen , Proc. Natl. Acad. Sci. USA 2018, 115, 909. 29339509 10.1073/pnas.1710874115 PMC5798324

[88] L. Csoka , I. C. Hoeger , O. J. Rojas , I. Peszlen , J. J. Pawlak , P. N. Peralta , ACS Macro Lett. 2012, 1, 867. 10.1021/mz300234a 35607134

[89] N. H. Langton , D. Matthews , Br. J. Appl. Phys. 1958, 9, 453.

[90] C. M. Lueng , H. L. W. Chan , C. Surya , C. L. Choy , J. Appl. Phys. 2000, 88, 5360.

[91] A. G. Chynoweth , J. Appl. Phys. 1956, 27, 78.

[92] L. A. Reznitchenko , A. V. Turik , E. M. Kuznetsova , V. P. Sakhnenko , J. Phys.: Condens. Matter 2001, 13, 3875.

[93] D.‐W. Fu , H.‐L. Cai , Y. Liu , Q. Ye , W. Zhang , Y. Zhang , X.‐Y. Chen , G. Giovannetti , M. Capone , J. Li , R.‐G. Xiong , Science 2013, 339, 425. 23349285 10.1126/science.1229675

[94] Y. Zhang , Y. Liu , H.‐Y. Ye , D.‐W. Fu , W. Gao , H. Ma , Z. Liu , Y. Liu , W. Zhang , J. Li , G.‐L. Yuan , R.‐G. Xiong , Angew. Chem., Int. Ed. 2014, 53, 5064. 10.1002/anie.201400348 24692257

[95] S. Horiuchi , Y. Tokunaga , G. Giovannetti , S. Picozzi , H. Itoh , R. Shimano , R. Kumai , Y. Tokura , Nature 2010, 463, 789. 20148035 10.1038/nature08731

[96] X.‐G. Chen , X.‐J. Song , Z.‐X. Zhang , P.‐F. Li , J.‐Z. Ge , Y.‐Y. Tang , J.‐X. Gao , W.‐Y. Zhang , D.‐W. Fu , Y.‐M. You , J. Am. Chem. Soc. 2019, 142, 1077. 31851495 10.1021/jacs.9b12368

[97] C. Wang , T. Xia , X. Lou , Ceram. Int. 2018, 44, 22053.

[98] W. Liu , X. Ren , Phys. Rev. Lett. 2009, 103, 257602. 20366285 10.1103/PhysRevLett.103.257602

[99] Y. Liu , Y. Chang , F. Li , B. Yang , Y. Sun , J. Wu , S. Zhang , R. Wang , W. Cao , ACS Appl. Mater. Interfaces 2017, 9, 29863. 28799748 10.1021/acsami.7b08160

[100] P.‐F. Zhou , B.‐P. Zhang , L. Zhao , X.‐K. Zhao , L.‐F. Zhu , L.‐Q. Cheng , J.‐F. Li , Appl. Phys. Lett. 2013, 103, 172904.

[101] Y. Guo , H. Fan , C. Long , J. Shi , L. Yang , S. Lei , J. Alloys Compd. 2014, 610, 189.

[102] A. Hussain , C. W. Ahn , J. S. Lee , A. Ullah , I. W. Kim , Sens. Actuators, A 2010, 158, 84.

[103] N. B. Do , H. D. Jang , I. Hong , H. S. Han , D. T. Le , W. P. Tai , J. S. Lee , Ceram. Int. 2012, 38, S359.

[104] X. Liu , H. Guo , X. Tan , J. Eur. Ceram. Soc. 2014, 34, 2997.

[105] M. H. Lee , D. J. Kim , J. S. Park , S. W. Kim , T. K. Song , M.‐H. Kim , W.‐J. Kim , D. Do , I.‐K. Jeong , Adv. Mater. 2015, 27, 6976. 26444562 10.1002/adma.201502424

[106] D. Lin , Z. Li , S. Zhang , Z. Xu , X. Yao , J. Am. Ceram. Soc. 2010, 93, 941.

[107] H. Tian , C. Hu , X. Meng , Z. Zhou , G. Shi , J. Mater. Chem. C 2015, 3, 9609.

[108] C. Hu , H. Tian , X. Meng , G. Shi , W. Cao , Z. Zhou , RSC Adv. 2017, 7, 7003.

[109] S.‐E. Park , T. R. Shrout , J. Appl. Phys. 1997, 82, 1804.

[110] T. He , Q. Shi , H. Wang , F. Wen , T. Chen , J. Ouyang , C. Lee , Nano Energy 2019, 57, 338.

[111] S. H. Shin , S. Y. Choi , M. H. Lee , J. Nah , ACS Appl. Mater. Interfaces 2017, 9, 41099. 29130682 10.1021/acsami.7b11773

[112] J. Zhou , Y. Gu , P. Fei , W. Mai , Y. Gao , R. Yang , G. Bao , Z. L. Wang , Nano Lett. 2008, 8, 3035. 18707178 10.1021/nl802367t

[113] N. I. Kim , J. Chen , W. Wang , M. Moradnia , S. Pouladi , M. K. Kwon , J. Y. Kim , X. Li , J. H. Ryou , Adv. Funct. Mater. 2021, 31, 2008242.

[114] J. M. Wu , C. Y. Chen , Y. Zhang , K. H. Chen , Y. Yang , Y. Hu , J. H. He , Z. L. Wang , ACS Nano 2012, 6, 4369. 22482745 10.1021/nn3010558

[115] E. S. Hosseini , L. Manjakkal , D. Shakthivel , R. Dahiya , ACS Appl. Mater. Interfaces 2020, 12, 9008. 32011853 10.1021/acsami.9b21052 PMC7146751

[116] S. Park , X. Guan , Y. Kim , F. X. Creighton , E. Wei , I. Kymissis , H. H. Nakajima , E. S. Olson , Trends Hear. 2018, 22, 1. 10.1177/2331216518774450 PMC5987900 29732950

[117] S. Ghenna , F. Giraud , C. Giraud‐Audine , M. Amberg , IEEE Trans. Ind. Electron. 2017, 65, 4880. 10.1109/TOH.2016.2607200 27623597

[118] Q. Xu , Y. Li , J. Dyn. Syst., Meas., Control 2010, 132, 041011.

[119] B. Chen , T. Li , Q. Dong , E. Mosconi , J. Song , Z. Chen , Y. Deng , Y. Liu , S. Ducharme , A. Gruverman , F. De Angelis , J. Huang , Nat. Mater. 2018, 17, 1020. 30250177 10.1038/s41563-018-0170-x

[120] S. E. Park , T. R. Shrout , J. Appl. Phys. 1997, 82, 1804.

[121] F. Li , L. Jin , R. Guo , Appl. Phys. Lett. 2014, 105, 232903.

[122] Q. M. Zhang , H. Li , M. Poh , F. Xia , Z. Y. Cheng , H. Xu , C. Huang , Nature 2002, 419, 284. 12239563 10.1038/nature01021

[123] M. Q. Le , J. F. Capsal , J. Galineau , F. Ganet , X. Yin , M. D. Yang , J. F. Chateaux , L. Renaud , C. Malhaire , P. J. Cottinet , R. Liang , Sci. Rep. 2015, 5, 11814. 26139015 10.1038/srep11814 PMC5155611

[124] Q. M. Zhang , V. Bharti , X. Zhao , Science 1998, 280, 2101. 9641912 10.1126/science.280.5372.2101

[125] Y. Liu , B. Zhang , W. Xu , A. Haibibu , Z. Han , W. Lu , J. Bernholc , Q. Wang , Nat. Mater. 2020, 19, 1169. 32601482 10.1038/s41563-020-0724-6

[126] C. Frias , J. Reis , F. C. e Silva , J. Potes , J. Simões , A. T. Marques , Compos. Sci. Technol. 2010, 70, 1920.

[127] M. Sebastian , J. A. Peters , C. C. Stoumpos , J. Im , S. S. Kostina , Z. Liu , M. G. Kanatzidis , A. J. Freeman , B. W. Wessels , Phys. Rev. B 2015, 92, 235210.

[128] J. Kang , L. W. Wang , J. Phys. Chem. Lett. 2017, 8, 489. 28071911 10.1021/acs.jpclett.6b02800

[129] D. B. Kim , K. H. Park , Y. S. Cho , Energy Environ. Sci. 2020, 13, 2077.

[130] K. Maity , S. Garain , K. Henkel , D. Schmeißer , D. Mandal , ACS Appl. Polym. Mater. 2020, 2, 862.

[131] S. Gong , B. Zhang , J. Zhang , Z. L. Wang , K. Ren , Adv. Funct. Mater. 2020, 30, 1908724.

[132] N. A. Hoque , P. Thakur , P. Biswas , M. M. Saikh , S. Roy , B. Bagchi , S. Das , P. P. Ray , J. Mater. Chem. A 2018, 6, 13848.

[133] L. Lu , W. Ding , J. Liu , B. Yang , Nano Energy 2020, 78, 105251.

[134] E. Kar , N. Bose , B. Dutta , S. Banerjee , N. Mukherjee , S. Mukherjee , Energy Convers. Manage. 2019, 184, 600.

[135] Y. Rakita , O. Bar‐Elli , E. Meirzadeh , H. Kaslasi , Y. Peleg , G. Hodes , I. Lubomirsky , D. Oron , D. Ehre , D. Cahen , Proc. Natl. Acad. Sci. U. S. A. 2017, 114, E5504. 28588141 10.1073/pnas.1702429114 PMC5514731

[136] L. Shu , S. Ke , L. Fei , W. Huang , Z. Wang , J. Gong , X. Jiang , L. Wang , F. Li , S. Lei , Z. Rao , Y. Zhou , R. K. Zheng , X. Yao , Y. Wang , M. Stengel , G. Catalan , Nat. Mater. 2020, 19, 605. 32313265 10.1038/s41563-020-0659-y

[137] S. Huang , L. Shi , T. Zou , H. Kuang , P. Rajagopalan , H. Xu , S. Zhan , J. Chen , W. Xuan , H. Jin , S. Dong , H. Zhou , X. Wang , W. Yin , J. M. Kim , J. Luo , Adv. Energy Mater. 2020, 10, 2002470.

[138] A. A. Khan , M. M. Rana , G. Huang , N. Mei , R. Saritas , B. Wen , S. Zhang , P. Voss , E. A. Rahman , Z. Leonenko , S. Islam , D. Ban , J. Mater. Chem. A 2020, 8, 13619. 10.1021/acsami.0c12874 32969216

[139] Z. L. Wang , Adv. Mater. 2007, 19, 889.

[140] J. H. He , C. L. Hsin , J. Liu , L. J. Chen , Z. L. Wang , Adv. Mater. 2007, 19, 781.

[141] S. Liu , L. Wang , X. Feng , Z. Wang , Q. Xu , S. Bai , Y. Qin , Z. L. Wang , Adv. Mater. 2017, 29, 1606346. 10.1002/adma.201606346 28218797

[142] Z. Zhang , Q. Liao , Y. Yu , X. Wang , Y. Zhang , Nano Energy 2014, 9, 237.

[143] J. Sun , Q. Hua , R. Zhou , D. Li , W. Guo , X. Li , G. Hu , C. Shan , Q. Meng , L. Dong , C. Pan , Z. L. Wang , ACS Nano 2019, 13, 4507. 30875189 10.1021/acsnano.9b00125

[144] R. Bao , C. Wang , Z. Peng , C. Ma , L. Dong , C. Pan , ACS Photonics 2017, 4, 1344.

[145] J. Nie , Y. Zhang , L. Li , J. Wang , J. Mater. Chem. C 2020, 8, 2709.

